# The development of clinical reasoning throughout the training and career of psychiatrists in Singapore

**DOI:** 10.5116/ijme.64d9.e64b

**Published:** 2023-08-31

**Authors:** Daniel Poremski, Kay Wee Kwang, Felix Rene Zhi Yuan Lim, Yiqing Yan, Giles Ming-Yee Tan, Kang Sim

**Affiliations:** 1 Institute of Mental Health, Singapore

**Keywords:** Decision making, clinical reasoning, psychiatry, medical education, reflectivity

## Abstract

**Objectives:**

The current study sought to explain how different professional experiences led Singaporean psychiatrists to alter their clinical reasoning processes as their careers evolved from psychiatry residents to senior consultant psychiatrists.

**Methods:**

The current qualitative study interviewed 26 clinicians at various stages of their psychiatric career, spanning residents to senior psychiatrists.  The authors used a constructivist grounded theory approach to structure the collection and analysis of data. Analyses produced a dense theoretical explanation rooted in the experiences of participants.

**Results:**

Several differences emerged between the way psychiatry residents and senior psychiatrists explained their reasoning process and the experiences on which they based their preference. Residents preferred using deductive logic-driven frameworks that were diagnosis-centric, because of the pressures they experienced during their training and assessments. Senior psychiatrists emphasized a more holistic and problem-centric approach. Participants attributed the changes that occurred over time to practical experiences, such as their greater clinical responsibility and independence, and individual experiences, such as growing sensitivity to the clinical reasoning process or their growing propensity for professional reflectiveness. These changes manifest as an increase in repertoire and flexibility in deployment of different clinical reasoning strategies.

**Conclusions:**

It is important for trainees to be aware of the deductive and inductive modes of clinical reasoning during supervision and to be comfortable with shifting clinical focus from diagnoses to specific individual problems. Training programs should provide and plan adequate longitudinal clinical exposure to develop clinical reasoning abilities in a way that allows consequences of decisions to be explored. Continued faculty development to ease the diversification of clinical reasoning skills should be encouraged, as should reflectivity in the learners during clinical supervision.

## Introduction

The importance of understanding and studying the clinician’s reasoning process has long been recognized^1^^-^^3^ and this extant literature has led to multiple improvements in the pedagogical methods deployed to enhance it.^4^^-^^6^ Many different approaches have been proposed to characterize the way in which people approach clinical reasoning. Several approaches share the same nomenclature (deductive and inductive logic) but structure the reasoning process differently. For example, some theorists have proposed a holistic approach that incorporates deduction, abstraction, abduction and induction.^7^ suggesting that clinicians use these methods in an iterative process to formulate the case, diagnose the condition and consequently recommend treatment.^7^  Whereas others differentiate the contribution made by each process to different stages of the clinical reasoning process.^8^ According to this latter theory, which follows Peirce’s seminal typology,^9^ when a clinician is presented with a new case that contains signs that fit with established criteria of a diagnosis, for example, deductive reasoning is used to produce a diagnosis. But if the case does not present with such elements, the clinician may use abductive reasoning, first making multiple hypotheses, and then selecting from the most relevant.^8^ In this paradigm, inductive reasoning (more akin to hypothesis testing) is predominantly used for helping clinicians choose appropriate treatment choices, but not generally for arriving at diagnoses.

Clinicians pull on sources of information in every instance and rely on them in defined ways.^3^ Regardless of the nomenclature used to describe the various processes, the finding that people use multiple methods, switching between them when presented with different cases, appears to be echoed through these schools of thought.^1^^,^^7^^,^^8^^,^^10^^,^^11^ These theories highlight the flexibility a physician demonstrated while reasoning, but treat these sets of skills as static, ignoring the fact that they may develop differently based on the physician’s experiences.

In psychiatry, arriving at specific diagnosis and management plan can be more challenging compared to other medical disciplines, given the lack of objective tests used to diagnose or prognosticate.  The discipline requires, more so than in any other, empathy and affability to engage the person and elicit the information required to inform the clinical reasoning process.^7^^,^^10^^,^^12^^,^^13^ Understanding that the person is more than the sum of their parts is crucial.^14^

To date, despite understanding the importance of clinical reasoning and its emergence during psychiatry residency training, extant studies mainly focus on ways of improving clinical reasoning and the methods used for testing it. ^4^^-^^6^^,^^13^ Few studies have attempted to explore how clinical reasoning evolves throughout the training and career of psychiatrists or the experiences that influence its evolution. These are important targets for future research as a greater understanding of these phenomena may inform and improve the pedagogical strategies used to foster clinical reasoning over the career of psyciatrists.^15^

Considering the sparse literature on examining the nature and development of clinical reasoning in the training and career of psychiatrists, the current study sought to determine how psychiatrists used different clinical reasoning processes as their careers evolved and to determine which professional experiences contributed to this evolution.

## Methods

To understand the way in which modes of clinical reasoning have changed over the course of the career of psychiatrists, we chose a qualitative approach.  Specifically, to develop an understanding rooted in the experiences of Singaporean psychiatrists, we chose a grounded theory methodology.^16^^,^^17^ This is a cyclical process in which a question is answered by means of iterative data collection, data analysis, data coding and theory building. It produces a dense theoretical account grounded in empirical data that can incorporate existing theory or be rooted entirely in a new one. While several theories exist to explain clinical reasoning, we adopted the idea that experience leads to changes in practice.  Our purposive sampling framework, described below, reflects this premise.^18^ and eschewed all other theories. The Institutional Research Review Committee and national Domaine Specific Review Board approved the study. We used the Consolidated criteria for reporting qualitative research to structure the manuscript.^19^

### Setting

Singapore is a small island nation with approximately 6 million inhabitants. Its healthcare system is a hybrid of public and private providers, but all residents are obliged to contribute to a personal government-managed account to cover costs of healthcare services. The Institute of Mental Health (IMH), where the study was based, acts as the main source of tertiary psychiatric care. IMH serves as one of the main national sites for Singapore’s The National Psychiatry Residency Program. This centralized postgraduate training program has followed the American Accreditation Council for Graduate Medical Education—International (ACGME-I) system since 2010. ^20^ Residents rotate across several training settings during their 5 years, covering different facets of psychiatry. To supplement its demand for mental health services, foreign staff are actively recruited, and so psychiatrists from multiple countries practice in Singapore.

### Participants and Recruitment

We operationalized, for recruitment purposes, experience as professional rank, rather than age. As such, we divided our sampling framework to include five career groups. Junior residents completed medical school and were in their first three years of training within the only national psychiatry residency program. Senior residents were in their last two years of their residency training and had rotated through several departments and psychiatric postings. Associate consultants were recently employed as fully independent psychiatrists and no longer received supervision. Consultants and Senior consultants had increasing years of experience. Consultants were usually promoted to senior positions after six years in the role. As noted below, the experiences of our participants led to a decision to abandon this grouping in favour of one which more parsimoniously explained the milestones in the development of clinical reasoning. We provide theory-relevant demographic details of the participants below. We identified potential participants via the hospital’s internal list of psychiatrists and recruited them by means of invitation email, as per the approved procedure. All potential participants approached agreed to participate.

We sought to interview 25 participants, targeting five in each of the five career groups. We proceeded by recruiting and interviewing members of our five groups sequentially, starting with the least experienced. Once one group was interviewed, we conducted preliminary analyses to develop a set of theories related to our main research interests, before moving on to the next group. The decision to limit each group to five stemmed from the limited size of the population from which to draw potential participants. We conducted one additional interview with the associate consultants because of a larger variety of opinions related to clinical reasoning amongst the group. Following the completion of the 26 interviews we were satisfied that conducting additional interviews would not add or alter our theory (saturation). Recruitment stretched from June 2021 to October 2021. All participants approached agreed to participate, likely facilitated by the alternative to conduct teleconference interviews at their convenience. All participants gave written informed consent prior to their participation in this study. No names were recorded to ensure anonymity. 

### Interviews (data collection)

Interviews were semi-structured and followed an interview guide containing five general topics, namely, context (participants training background and experience with psychiatry), clinical reasoning definition, the development of clinical reasoning, an example of the clinical reasoning process, and the training of clinical reasoning skills. The interview guide can be found in the online supplement material. The first author conducted all the interviews alone. Interviews lasted an average 64 minutes (standard deviation 8 minutes). Of the 26 interviews, 24 (92%) were conducted via teleconferencing platforms, and the remainder were conducted in person in the offices of participants. Participants spoke one average 75% of the time (standard deviation 7.4%). At the end of each interview, the interviewer took notes about the way each interview contributed to the emerging theory and reflected upon the degree to which participants had volunteered information and the rapport he had built with participants. These notes were used in the group discussions mentioned in the analysis section below. Content related to rapport was used to monitor the robustness of the digital data collection process. Rapport was especially strong amongst consultants and senior consultants, with which the interviewer had previous working relationships. Three participants took time to warm up to the experience of participating in qualitative interviews. Most participants would have had experience with the teleconferencing platform in their daily duties.

While the rank order in which interviews were conducted made it impossible to validate content that emerged in later interviews during earlier interviews, we anticipated this limitation as an eventuality. We mitigated this by asking participants of early interview to reflect on the way in which they believed senior psychiatrists reasoned. This allowed us to discuss modes of reasoning that were not necessarily their own, but modes of reasoning they encountered in others (who were often their superiors). While these discussions could only be conducted theoretically to a large extent, they provided some means by which modes of reasoning that only emerged in first-hand accounts in later interviews could be discussed in earlier interviews. This concerned mostly modes of reasoning that heavily relied on intuition and pattern recognition, which juniors felt their superiors engaged in more frequently than deductive modes. To mitigate the impact of this choice, we used primacy to assign importance to content during the analysis, as described below.

### Data analysis

Previous theories exist to approach the data in a deductive process, however, given the multitude of clinical reasoning processes, and the known propensity for clinicians to adapt different styles over time and between cases, we chose to employ a predominantly inductive approach to coding the data. ^16^ As such, we did not set an a priori conceptual framework into which content would be divided but wanted to arrive at that process in a data-driven manner.^17^ Our process, therefore, follows a grounded theory approach^21^ in which emerging theories altered the line of questioning of subsequent interviews. This process suits the objectives of our project to develop a local theory of how clinical reasoning emerged in junior clinicians and evolved into the way senior clinicians practiced.^17^

The second, third and fourth authors transcribed the interviews verbatim, with supervision of the first, shortly after interviews were conducted. The first author coded the interviews with the direct support of the fourth author to arrive at a list of extensive codes that attempted to separate any potential ideas or directions to open several lines of inquiry.  We reconstructed the content by means of frequent internal discussion over the course of the interview/ analysis process. These discussions led to notes, or memos, which documented the way team member understood and interpreted the content of the interviews. This oral process allowed the opinions of the team members to be shared and consolidated to direct future questions and structure the theory.

The initial interviews with junior staff produced significant content related to modes of clinical reasoning. While further interviews with senior psychiatrists were not limited to these initial modes of reasoning, we did prompt participants to talk about them if they omitted a mode mentioned in earlier interviews. What emerged as a result of this process was a discussion of clinical reasoning modes and the way practitioners changed with time. We cross-coded all general elements related to changes in practices (termed evolution), with modes of reasoning. We then performed a selective coding pass over the data and focused on the core changes once all interviews were completed. During this pass, we focused on the way in which modes of reasoning changed and the mechanisms that led to change. Finally, the team attempted to organize the mechanisms that led to evolution in a way that reflected the participants’ explanations.

We assigned importance to the content by considering its primacy during the interviews, with a greater priority given to modes of reasoning and reasons for changing that surfaced early and without prompting. We also considered intensity, with greater importance given to content that was discussed at greater length. The analytic process did not seek to uncover all differences that existed between the professional grades, but rather focused on the clinical reasoning process and its change over time. NVIVO 11 software help us code and analyze content.

### Reflexivity

The first author acted as the sole interviewer. He has extensive experience interviewing psychiatrists from Singapore and has experience with the local idioms and creole.   He also has extensive experience in psychiatry.  The two senior authors are senior psychiatrists who lead psychiatry educational program for undergraduate and postgraduate learners in psychiatry. They were not involved in data collection to limit the impact of preconceptions about the process. Given their positions in the leadership of educational services, the team purposefully avoided potential censorship of content related to shortcomings of the education program during the analysis. Because all members of the team participated in the discussion of content and contributed to the way in which our theory developed, it is not entirely possible to divest preconceptions about clinical reasoning development from our process. However, the range of previous experience with clinical reasoning theories spanned the naïve to the experienced, and the first author was mindful to ensure emerging ideas were given full consideration to weigh their merit in a data-driven manner and avoided dismissing content that did not fit with known theories of clinical reasoning.

## Results

The evolution of clinical reasoning accelerates with career maturity especially at the transition of resident to independent psychiatrist. As such, we revised our way of grouping data and dividing the participants between these two states. We have elected to present the findings along this dichotomy. The original sampling framework divided by five ranks was abandoned, but we kept the data needed to provide fine distinction between ranks of independent psychiatrists.

Three participants received their training in Australia, two had training in the UK, two had trained in India and one in Malaysia. The remaining (n=18) had been trained solely in Singapore under a system that follows ACGME-I. 20 Eight of the 26 participants were women.  Quotes provided below are tagged with a numeric participant ID, professional rank, and location of medical training, to contextualize their comments. They are presented verbatim.

### Residents

#### Modes of clinical reasoning

Residents-in-training tended to use structured frameworks to guide their interviews and clinical reasoning, mentioning often diagnostic classification frameworks, such as Diagnostic and Statistical Manual (DSM). They explained that they drew their information from service users, their informants, existing medical documentation, colleagues and superiors, and medical literature. The process was highly analytic, and they relied on rote knowledge to arrive at a decision that had to be defensible. The consideration of defensible decisions led the residents to be explicit about which elements of a case led to their conclusions. As their clinical decisions often had to be vetted by senior consultants, they rely less on intuition to guide their decision, but when they did, intuition drove the way they distinguished one diagnosis from another.

#### Factors influencing change of clinical reasoning over time

Residents in training linked the way in which their clinical reasoning process developed to the assessment structure of their residency training. The focus on diagnosis comes ostensibly from case-based training paradigm. In addition, the diagnostic, treatment, and prognostic considerations needed to successfully clear summative exams heavily influenced their thought process. Residents felt the pressure to arrive quickly at a diagnosis and management plan within the space of a single consult because it was under such scenarios that their proficiency was graded.

“I would say that I transitioned more out of necessity than out of preference that I think it’s easier to do it. I think what happened was when we do the intermediate exams, which is the UK membership for the Royal College of Psychiatrists, you have to go through the OSCE stations right, the structured clinical interviews. And you are required to only ask enough to establish the diagnosis, but not the full criteria set. Otherwise, you would have no time. So, once you have established enough criteria to make the diagnosis, you move on to your next line of questioning.” 21013 associate consultant, training in Singapore

They noted that time constraints may lead to a greater reliance on intuition, but the degree to which they felt confident in this strategy was low. This changed as seniority was recognized, as described below.

“I think if everyone had more time or had less patients each slot, clinic slot, most people would actually like to find out more rather than jump straight into continuing the treatment, continuing the diagnosis, rather than questioning what some of the parts are that do not really gel.” 21002 resident year 3, training in Singapore

### Independent psychiatrists

#### Modes of clinical reasoning

Senior consultants appeared to be more fluid in the frameworks they used to structure their clinical reasoning process. Rather than following diagnostic classification frameworks, they stated using a variety of frameworks, including biographical^22^ and ecological structures.^23^ They drew information from sources like those of their juniors. However, none noted relying on information drawn from textbooks. Another difference related to the way in which they drew information from their own reactions to patients. This process emerged from their understanding of transference and countertransference, in that, the responses of their patients based on past encounters may influence their own response which can be related to their own earlier encounters as well. They appeared to be adding to their repertoire of strategies rather than dispensing with one in favour of another. Most acknowledged that the analytic approach was very sensible, but they also relied on inductive forms of reasoning to determine what might be important for their current case.  They placed less importance on diagnosis, recognizing that it may shift with time, may not immediately provide a solution to the patient, and may take multiple interactions to reach sufficient trustworthy information to generate the diagnosis. They preferred to understand the immediate needs or concerns of the patients and observed that diagnostic labels and premature treatment recommendations may be antagonistic to the patient seeking support:

“And sometimes people do not like it when you give them solutions that are not fitting with their kind of profile.” 21026 senior consultant, training in the UK

“So don’t get me wrong the psychiatric residency gives us the basic building blocks to be a good psychiatrist,  we are exposed to clinical psychiatry , we are exposed to getting a good history, we are exposed to psychology and we also do psychotherapy, but with time, it is an amalgamation  of all these skills, which comes really from working with people, many patients, and the realization that all this are just the very basic tools that are given to us, and how we use them becomes important, and using the more sensitivity approach to find out more about the person actually comes with realization of what the patient needs through all my working experiences with them.” 21024 senior consultant, training in Singapore

“INTERVIEWER: to put this squarely on the topic of clinical reasoning, what is the benefit of having that perfect puzzle fit [rapport] when it comes to your overall goals of clinical reasoning and decision making. What is the benefit?”

PARTICIPANT: the benefit is the patient comes back. Because we are in chronic disease management. It doesn’t… you know the duration of untreated psychosis (DUP) for some of the diseases, DUP or symptoms can be months or years. Another week of not taking the medicine will not make a difference so what is important is that the patient comes back you see. […] Because you see at the end of the day people come to us, many of them have suffered, and many of them have suffered and many of them don’t have the opportunity to offload all their angst. So, if you somehow manage to tune into correctly and this person suddenly they unload this floodgate of emotion “wow” at the end they feel “I had a good experience at the psychiatrist today”. 21022 senior consultant, training in Singapore

They accept that the working alliance needed for patients to follow treatment plans may take time to build. They recognized that rushing into recommendations and prescriptions without first understanding patient preferences might lead to transient commitments from the patient. They recognized the value that came from building a longer-term relationship as such efforts lay the foundation for continuity of clinical reviews and treatment adherence once the person has left the clinic. As such, these elements took priority over thinking about where the service user fits into existing diagnostic frameworks. With their seniority came the understanding that diagnoses may change with accrual of added information:

“I think I would have liked to have been told that we should be open to revising diagnosis, because I find that it is a bit artificial in a limited consultation that you, especially if you only have 30 minutes, or you sometimes have even less, to necessarily rush into a diagnosis when you can, sometimes setting a diagnosis is like summarizing the person’s condition for the last 20 or 30 years, or whole life […]  I don’t think, I don’t really remember being told or taught that along the way we want to be open to revising the diagnosis, as and when we have more information coming, […] I mean making a diagnosis is important, but at the same time, it is important to be open that with more information, we may want to accept that we can revise the diagnosis. And revision of the diagnosis is not an admission of “oh we are poor clinicians” but it is actually a mark that we are prepared to learn because of our journey as a clinician.”  21025 senior consultant, training in Singapore

The degree to which they confirmed employing clinical intuition varied. Some believed that its use should be bridled, whereas others felt it was an unavoidable part of the way experience accumulates and is employed.

“Because as they [referring to her colleagues] progress to a mastery level, expert level, they know so much that it’s really at an unconscious level – they don’t have to verbalise it, they don’t have to document it as such. But they just know. So, they may say that it’s gut feel, but I would contend that it’s actually years of experience and clinical reasoning that led them to that intuition. It’s not really intuition, just that they had lost the skill to elaborate because they are such masters in their craft.” 21018 consultant, training in Singapore

What emerged as a relatively stable commonality amongst independent psychiatrists was that they used their intuition predominantly to increase their index of suspicion when cases were more complicated.  Unlike residents, they did not affirm relying on this strategy to arrive at diagnoses.

“So, you need to be careful about when you use it. You should never use gut feeling to dictate risk assessment. […] So broadly you should never use gut feeling to not intervene, or not treat a patient. But you can use gut feeling to err on the side of caution. And just be a little but more careful about your management. So, I think personally that is what we should be using gut feeling for.” 21020 consultant, training in Singapore

Factors influencing change of clinical reasoning over time

Several factors lead to independent psychiatrists to change in the way they reasoned clinically over time. Based on the primacy of our participants’ responses and the length at which they discussed these factors (intensity) we highlighted the role of changes in duties and responsibilities, the sensitivity to the clinical reasoning process and biases, and the conscious shifting of ruminations to more reflective exercises.

#### Change in duties

For independent psychiatrists, several factors contributed to the way in which they changed their clinical reasoning processes. Namely, the transition to being wholly responsible for a case and its outcomes led to the alteration of their priorities. This change in duties required a different clinical reasoning approach, one which was more likely to see the patient as a person with problems, rather than ticking the checklist to fit a diagnostic label. The decision to focus less on diagnosis might stem from a greater sensitivity to the time rapport may take to develop, with senior clinicians often placing themselves in the shoes of their new patients and considered whether the patients would feel comfortable sharing their issues at the first meeting:

“Look, I’m a stranger to the patient, the patient walks into my room for the first time, and in fact, if I put myself in the patient shoes, then I [the patient] have to go and talk to a psychiatrist. I’m literally opening up myself completely to a stranger, so it’s literally laying out my life, so there is that sense of vulnerability, especially in patients in psychiatry, so if I [the psychiatrist] am not able to achieve a trust or rapport in those first few minutes of the patient coming in, I can forget about you know, clinical formulation, clinical reasoning.” 21016 consultant, training in the UK

They also place greater importance on getting the patient to return and reliably engage with the process of consultation rather than worry about immediately addressing and solving concerns. The decision to focus more on momentary problems stems in part from the greater length of time they have had to get to know cases, as they often kept the same caseload over time.

The benefit of being able to follow cases over a longer period emerged with seniority. Participants noted that they were sometimes blind to the subsequent development of cases they had seen during their training rotations because they lost access to the patients once they rotated out.  Senior consultants noted that without this feedback loop, which could be used to validate or correct the decisions they had made over the course of their management of the case, they were less able to build confidence in their clinical reasoning process. 

“We can't help it because that's what medicine is about - It is a lot of mystery, a lot of unpredictability about it, and a person who seems to be like really doing well on the day, the next day things can change for them. So yeah, but as much as possible, when somebody has kind of like left some kind of questions in me, I will go back to check. I know it's not in the protocol, it's not in the curriculum, it's not in our daily work routine but I do it on my own. It's actually out of my own interest I do it. […] I do it because I want to know what happened.” 21014 consultant, training in India

“Especially if you treated them for many years, because they would have gone through several relapses with you and you kind of know they really need the 5 milligrams of olanzapine, and it is non-negotiable, “every time I drop below 5 you have a relapse” … that sort of thing. So, after a while, you kind of know how that patient, what they need, though, that factors into the decision-making process.” 21022 senior consultant, training in Singapore

Being in a position to follow cases for longer periods of time also allowed them to observe the impact of their choices and adjust their reasoning when they saw similar cases in the future. Residents did not express this experience, likely because they had not fully experienced and appreciated the impact that longitudinal access to cases could have on their thought process.

#### Sensitivity to clinical reasoning

Another element that contributed to the change in clinical reasoning was the greater sensitivity of the clinical reasoning process itself and awareness of the common pitfalls clinicians encounter because of the process of practice. While many participants spontaneously noted that they had not given much thought to their clinical reasoning process, those who had contemplated the process previously noted the challenges. For example, once the interviewer probed the challenges, many recognized primarily the challenge of biases, especially when arriving at treatment choices. Bias may interfere with what clinicians think might help the person, for example if a clinician thinks a certain action might help, they may inadvertently seek information that confirm the decision to follow that course of action.

“So, let’s say at the onset we have made the decision that somebody, if we are not conscious of it, we may end up reading the things in a way we might ordinarily not have, because we think somebody has a certain condition, then we start picking up the cues, and the confirmation bias.” 21025 senior consultant, training in Singapore

While confirmation bias is well known on an intellectual level, during time-sensitive consultations, it may be harder to differentiate bias from a decision firmly rooted in available evidence. The impact of countertransference was also described as an element to consider as it could allow reflection about the response of the clinician as well as influence of past encounters.

“And I guess because in the field of psychiatry, we do talk about our counter transference as well, so especially in identifying personality disorders or personality traits, then the counter transference is very useful guide. So, I do think there is a role for it. I don’t know if it can be taught, but certainly it can be honed over time.” 21019 consultant, training in Singapore

This sensitivity and awareness of the caveats of the various approaches led them to be mindful of the choices they made, such as in the way in which gut feeling was adapted to shift from optimizing diagnostic decisions under time limitations to increasing the index of suspicion in cases whereby a gut feeling might lead the psychiatrist to probe more extensively in certain directions.

### Shifting from Rumination to Reflection

Rumination occurs when a clinician experiences intrusive thoughts about a case they had seen, usually after working hours. Participants recognized the potential hazard of being caught up by the particulars of challenging cases. It could impede the cognitive process if they were just focused on one element of a case at the expense of other pertinent sources of information. However, they also recognized rumination’s importance in building a clinician’s clinical reasoning skill.

“I am ruminating whether I was ruminating or whether it was for clinical reflection... I would say it is very tough to, at least for myself to tease them out, because the rumination, in that sense, does contribute to the reflective piece, but I guess what separates the two is that rumination is a little bit purposeless, it is supposed to be purposeless, it is just thinking about it just because you had an unpleasant encounter which cases some distress and you just think about it, but you are not being mindful about why you are thinking about it. So, I do ruminate, but when I realize that I am ruminating I will make it a point to turn it into a reflection, and that is when there will be very clear reasons as to ‘ok I am thinking about this now’, and I would approach it in a more structured way, as to what has gone wrong, what has gone right.” 21020 consultant, training in Singapore

Participants believed it was mostly junior clinicians who expressed traits of perfectionism. They believed it to be a phase of clinical which passed more quickly with experience because diagnostic perfection was eschewed as the ultimate aim. Independent psychiatrists adapted the process of rumination to rather reflect upon their management choices:

“I think especially when I feel like I’m not sure what the next step should be or what the treatment is, it [treatment] is not really working, what should I do next, then I start to ruminate more.” 21019 consultant, training in Singapore

## Discussion

Our study sought to determine how psychiatrists used different clinical reasoning processes as their careers progressed and to determine which professional experiences contributed to the change. There were severable notable findings. First, psychiatry residents in training adopted a more deductive logic driven framework in their clinical reasoning, are more diagnosis centric as opposed to more senior clinicians who tended to emphasize a holistic perspective, and problem centric reasoning strategies.  While we approached the way in which psychiatrists changed their clinical reasoning process as an evolution of skills, it may be more accurate tocharacterize the phenomenon as an increase in repertoire and flexibility in deployment of range of clinical reasoning strategies.^3^ They do not dispense with types of clinical reasoning frameworks, but rather shift their preference to those that suit their style and focus. Second, practical (such as greater clinical responsibility) and individual (such as sensitivity to clinical reasoning process, reflectivity) factors influenced the change of modes of reasoning over time.

We have found, as others have documented, younger psychiatrists rely more on an analytic and deductive logic reasoning based on rote knowledge, while more experienced psychiatrists rely more on intuition and inductive logic to guide their clinical reasoning processes.^10^ This reliance can be related to the pedagogical framework in that there is a need to clearly provide justification for their decisions during the regular clinical supervision as well as during formative and summative assessments. Although intuition was a mode of clinical reasoning in more senior clinicians, intuition if used alone was recognized as a potential issue when it is used predominantly at the expense of other collected information. ^24^ It is thought that when such clinical intuition is carefully dissected or parsed, the source and components of these clinical instincts may be scrutinized and evaluated for the benefit of subsequent clinical reasoning.^25^As noted elsewhere, our independent psychiatrists used their gut feeling to increase their index of suspicion.^26^

The use of various types of decision-making strategies has long been recognized.^1^^,^^7^^,^^8^^,^^27^ The shift between them may be traced to changes in context that limit the utility of deductive approaches.^11^ What our findings add to this body of literature is the way in which the clinical focus contributes to the overall shift in use of inductive processes. While the corpus of knowledge clearly suggest training program may facilitate the acquisition of clinical reasoning skills by exposing trainees to a multitude of methods without prioritizing a particular method of clinical decision making, our findings push this widening of approaches further by suggesting that relaxing the emphasis on diagnosis during training might help junior clinicians broaden the diversity of their clinical focus, at least in psychiatry.

The factors that lead to the changes seen over time predominantly concern changes in duties and responsibilities (practical reasons), and a greater understanding of the clinical reasoning process and shifting ruminating thoughts to reflective exercises (individual reasons). Following up on patients under their own care over time with better knowledge of these individuals helped them shift the focus of their clinical meeting from one that prioritized arriving at a diagnosis and treatment alternatives to one that prioritized the person holistically and their current problems, as well as the bond that could support greater analytic specificity in the future. This shift led them to favour an inductive reasoning process that sought to observe the individual rather than apply a known construct to their experiences at the start. While seeing the person as a whole is a routine element of the curriculum,^28^ this has often been paired with the biopsychosocial model, which underscores the importance of consideration of predisposing, precipitating, perpetuating and protective factors within a case formulation framework. Independent psychiatrists in our study may move between such a formulaic approach to taking a more inductive or intuitive approach based on the current presentation of the person.

The observation that independent psychiatrists used counter-transference and their own emotional state as a source of information adds to the literature on use of intuition and its source.^26^ While the physical sensations associated with the “gut feeling” that arise from an incongruence between the presented information and the cognitive schema represent a well-documented use of emotions in clinical reasoning, our addition of harnessing countertransference goes beyond the current understanding. The phenomenon does not simply signal to a psychiatrist that something is amiss in their reasoning process but may provide information related to the patient under assessment.

The role of deliberate reflection has been empirically linked to improved reasoning skills,^29^ and as an exercise encouraged during formative years that then continued over time.^26^ Of note, there may be variations in which more junior learners understand the role of reflection and the distinction between deliberate reflection and rumination, with some independent psychiatrists noting that rumination may be less constructive because of its lack of process. It is also noteworthy that finding a conclusion to the reflective process was more beneficial compared with purposeless ruminative thought processes. While the benefits of deliberate reflection are clear^29^ it may be worth exploring further the way that ruminative thoughts can be better steered or guided towards reflective processes in clinical decision making.

### Implications

Based on our findings, there are several implications for the training of clinical reasoning in our psychiatry residents for the learner, program and faculty which may help other programs.

For the learner, there is a place to share these findings of the types of clinical reasoning seen amongst residents in training versus independent psychiatrists in terms of the shift from deductive to more inductive logic-based reasoning. This can raise awareness of the nature of such reasoning moving from a top down (starting from a premise such as knowing the diagnostic classification system and then using details to affirm or refute the specific diagnosis) to a more bottom-up logic-based reasoning (starting from clinical details elicited and observed during the clinical encounters and then arriving at an appropriate diagnosis). This sharing can facilitate better understanding and internalisation of the mental models of these different modes of clinical reasoning amongst the learners.^5^^,^^30^

For the program, there is a need to further individualise and integrate the learning longitudinally by careful and coordinated planning of adequate clinical exposure over the course of training. The longitudinal exposure with clinical responsibility at appropriate developmental stages over the course of training as found in this study can allow for inculcation of more inductive clinical reasoning framework that takes into account holistic perspective of the person^14^ with focus on personalized care and attention to presenting problems.

For the faculty, there can be greater emphasis on reflectivity about the learner’s clinical reasoning as the learner goes through training. In the process of supervision during direct observation and feedback, case-based discussion and case formulation^31^ the clinical supervisor can also share his own clinical reasoning model so as to make the implicit explicit.^6^ This can be supported by faculty development to enhance the knowledge and skills of the faculty in helping the development of range of clinical reasoning modalities within the learner over time.   

### Limitations

It is possible that the link we have made between experience and the modes of clinical reasoning of the various cohorts of psychiatrists may not be solely due to time spent in the profession but may partially be an artefact of their prior training. Teaching paradigms change. Such changes may explain why senior consultants thought in a different manner. This potential misattribution is tempered by the fact that the participants expressed that their years of experience accounted for their clinical reasoning approach.

Additionally, our classification of experience based on rank might be influenced by age and personal life experience. Some participants who entered psychiatry as a second career may have more years of life experience compared to younger psychiatrists who have relatively fewer years of experience. This challenge highlights the importance of life experience and maturity, which is important in the selection of psychiatry residents in Singapore.^20^ It is also relevant to note that the success of clinical reasoning processes was not evaluated by the authors, and therefore, we have assumed that proficiency accrues with experience. This also engenders the assumption that independent psychiatrists had superior, and therefore preferable, clinical reasoning practices.

Finally, our sampling strategy, which progressed by ascending cohort maturity, might have limited the degree to which content emerging exclusively amongst senior clinicians could be validated amongst other cohorts. As noted in our description of our interviews, we mitigated this limitation by allowing participants to talk about the modes of clinical reasoning they perceived in their senior colleagues. Participant checking may have been an alternative route to overcoming the limitations of the sampling framework.

## Conclusions

Our psychiatrists employed a greater variety of modes of clinical reasoning as their career developed. Participants mostly relied on deductive logic and rote learning driven patterns of reasoning in their earlier years of training before transitioning to patterns that favoured inductive logic and observation-driven approaches later in their career. Of note, early career psychiatrists focused on diagnosis, preferring diagnostic classification frameworks, such as the DSM, to structure their thinking. Experienced psychiatrists preferred to focus on present patient concerns and management plans, hence eschewing a diagnosis centric focus in favour of a more holistic problem-oriented approach. The change in modes of clinical reasoning seemed to be influenced by practical and individual factors, such as clinical exposure and reflectivity, which are amenable to support during the formative postgraduate training years.

### Acknowledgements

The authors would like to thank the participants for volunteering their time. We appreciate the candour with which they spoke to us.

### Conflict of Interest

The authors declare that they have no conflict of interest.
